# Toxicities of and inflammatory responses to moxifloxacin, cefuroxime, and vancomycin on retinal vascular cells

**DOI:** 10.1038/s41598-019-46236-2

**Published:** 2019-07-05

**Authors:** Hitomi Miyake, Dai Miyazaki, Yumiko Shimizu, Shin-ichi Sasaki, Takashi Baba, Yoshitsugu Inoue, Kazuki Matsuura

**Affiliations:** Division of Ophthalmology and Visual Science, Faculty of Medicine, Tottori University, Department of Ophthalmology, Nojima Hospital, Tottori, Japan

**Keywords:** Infection, Antimicrobials

## Abstract

Prophylactic intracameral injection of antibiotics is commonly used to prevent endophthalmitis after cataract surgery. However, devastating visual complications have been reported including hemorrhagic occlusive retinal vasculitis (HORV).To determine the toxic and inflammatory effects of moxifloxacin, cefuroxime, and vancomycin on human retinal vascular cells, human retinal vascular endothelial cells (RVEC) and pericytes were exposed to three antibiotics, and the adverse effects were assessed by membrane damage, loss of intrinsic esterase activity, kinetic cell viability, and inflammatory cytokine secretion. Their retinal toxicity was examined by live/dead assays after an intravitreal injection of the three antibiotics into mice eyes. In vascular cells in culture, membrane damage and loss of esterase activity were induced after exposure to the three antibiotics. The toxic effects were most obvious after moxifloxacin (RVEC, ≥125 μg/mL; pericytes, ≥1000 μg/mL) at 24 h. Cefuroxime also reduced esterase activity and the membrane integrity of vascular cells but were less toxic than moxifloxacin. Kinetic cell viability testing showed that 500 μg/mL of moxifloxacin exposure induced significant decrease (29%) in the viability as early as 1 h. When the inflammatory effects of the antibiotics were examined, a significant induction of IL-8 was observed especially by RVECs after exposure to cefuroxime or vancomycin which was exacerbated by L-alanyl-γ-D-glutamyl-meso-diaminopimelic acid (Tri-DAP), a NOD1 ligand. Intravitreal injections in mice showed that cefuroxime and vancomycin caused retinal and vascular toxicity extending to the inner nuclear layers. Collectively, moxifloxacin causes immediate damage to retinal vascular cells *in vitro*, while cefuroxime and vancomycin induced significant inflammatory effects on vascular endothelial cells and caused retinal toxicity. Surgeons need to be cautious of the toxicity when antibiotics are used prophylactically especially by intravitreal administration.

## Introduction

Endophthalmitis after cataract surgery is rare with an incidence of 0.014% to 0.2%^1–6^. Efforts to lower the incidence have been successfully attained by the use of intracameral antibiotics. Now, prophylactic intracameral injection of antibiotics is commonly used worldwide to prevent the endophthalmitis. The use of intracameral antibiotics is known to reduce the incidence of endophthalmitis by 6 to 22 fold^7,8^, and this was corroborated by a recent meta-analysis^9^. However, devastating visual complications have been reported including hemorrhagic occlusive retinal vasculitis (HORV), following the use of an intracameral injection of antibiotics. HORV is rare, however its visual outcome is devastating, and the prognosis is very poor. Despite this possibility, it is expected that prophylactic intracameral antibiotics will continue to be used based on the needs of the surgeons and the surgical setting.

In routine cataract surgery, the most widely used intracameral antibiotics are moxifloxacin, cefuroxime, and vancomycin. In the 2014 survey of the American Society of Cataract and Refractive Surgery members showed that each antibiotic was approximately equally used; moxifloxacin by 33%, cefuroxime by 26%, and vancomycin by 22%^10^. The concentration for the intracameral use ranged up to 1500 μg/ml for moxifloxacin and 3000 μg/ml for vancomycin and cefuroxime^11^. To have benefits without risking visual complications, the selection of the specific antibiotic and the concentration are important. However, the concentrations appear to have been empirically determined, and their safety profiles have not been thoroughly determined.

HORV has been shown to be associated with the use of vancomycin or cefuroxime, however the incidence of HORV is extremely low, and the exact mechanism for its development has not been determined. In addition, it cannot be ruled out that other intracameral antibiotics might induce HORV-like vascular endothelial damage under certain surgical settings.

To understand the retinal toxicity of antibiotics, ERG recordings and histological examinations of the retinal pigment epithelial (RPE) cells have been performed^12^. Before the recognition of HORV, the importance of retinal vascular endothelial cell as the presumed target of the antibiotics has been largely ignored.

Thus, the purpose of this study was to determine the effects of vancomycin, cefuroxime, and moxifloxacin on the retinal vascular endothelial cells and pericytes. To accomplish this, human retinal vascular endothelial cells (RVECs) or pericytes were exposed to moxifloxacin, cefuroxime, or vancomycin. Their direct toxicity was determined on cultured RVECs and pericytes, and their retinal toxicity was assessed by intravitreal injections into mice eye, Because HORV has been suggested to be mediated by immune-mediated tissue reactions, we analyzed how each antibiotic stimulates inflammatory cytokine secretion which could then prime presumed auto-inflammation or hypersensitivity reactions. Our results showed that moxifloxacin can cause significant vascular cell damage as early as 1 hour after application, however cefuroxime and vancomycin induced extensive retinal toxicity and primed strong IL-8 induction by the vascular endothelial cells.

## Results

### Comparisons of toxicity of moxifloxacin, cefuroxime, and vancomycin on human retinal vascular endothelial cells (RVECs) and pericytes *in vitro* measured by EthD-1 uptake

To determine whether moxifloxacin, cefuroxime, and vancomycin were toxic to RVECs and pericytes, we examined whether the cell membranes were damaged by the antibiotics. To do this, we used the degree of uptake of EthD-1 which is increased when the cell membrane is damaged.

**Figure 1:**
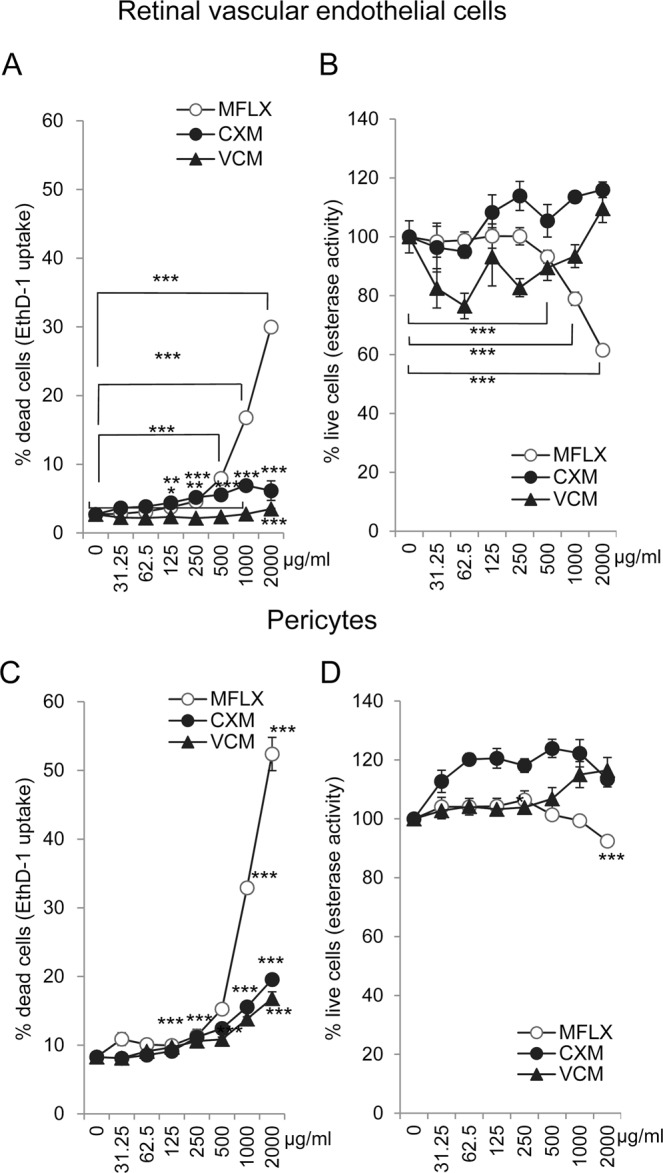
Effects of moxifloxacin, cefuroxime, or vancomycin on the integrity of the cellular membrane and viability of the cell *in vitro*. (**A**,**C**) Human retinal vascular endothelial cells (RVEC) and pericytes were exposed to the three antibiotics in serum-free media at the indicated concentrations and assessed for cell membrane damage using the uptake of ethidium homodimer-1 (EthD-1) at 24 h. (**B**,**D**) Cell viability was assessed by measuring the intrinsic esterase activity of the cells at 24 h. The esterase activity was significantly decreased with ≥1000 μg/mL of moxifloxacin. *P < 0.05; **P < 0.005; ***P < 0.001 by ANOVA and post-hoc test. N = 5. Similar results were obtained after repeated experiments.

Exposure of RVECs to moxifloxacin led to cell membrane damage, and the degree of damage was dose-dependent (Fig. 1A). More specifically, a significant increase in the cell membrane damage was observed with 125 μg/mL of moxifloxacin (*P* = 0.004), and 30% of the cells were damaged by 2000 μg/mL of moxifloxacin (*P* < 0.001). Cell membrane damage was also observed with 125 μg/mL of cefuroxime (*P* = 0.03), however, the degree of toxicity did not increase significantly with 2000 μg/mL of cefuroxime. In contrast, vancomycin had a minimal effect on cell membrane damage at 2000 μg/mL (*P* < 0.001).

Exposure of pericytes to these antibiotics also had similar damaging effect on the cell membranes (Fig. 1C). A significant increase in the cell membrane damage was observed with 1000 μg/mL of moxifloxacin (*P* < 0.001), and the damage reached 50% of the cells with 2000 μg/mL of moxifloxacin (*P* < 0.001). Cell membrane damage of pericytes was observed with 250 μg/mL of cefuroxime (*P* < 0.001) and 1000 μg/mL of vancomycin (*P* < 0.001)

Thus, moxifloxacin was more toxic, and higher concentrations, e.g., >500 μg/mL, increased the percentage of vascular cells whose membrane was damaged.

### Toxicity of moxifloxacin, cefuroxime, and vancomycin determined by intrinsic esterase activity

To evaluate a different aspect of toxicity of these antibiotics, we determined the cell viability by assessing the intrinsic esterase activity of cells which is abolished when the cells die. The intracellular esterase activity was measured using calcein AM as an esterase substrate, and a reduction of esterase activity indicated a decrease of cell viability (Fig. 1B,D). The results showed that exposure of RVECs to ≥500 μg/mL of moxifloxacin induced a significant decrease of esterase activity compared to no antibiotics exposure (Fig. 1B, *P* < 0.001). For cefuroxime and vancomycin, a dose-dependent decrease of esterase activity was not observed (Fig. 1B).

Exposure of pericytes to these antibiotics also showed that there was a significant decrease of esterase activity with ≥2000μg/mL of moxifloxacin (Fig. 1D, *P* < 0.001). Cefuroxime and vancomycin did not show appreciable reduction of esterase activity.

Collectively, ≥500 μg/mL moxifloxacin significantly reduced the viability of vascular cells as assessed by measurements of the cell membrane damage or esterase activity. This effect was more notable for RVECs than pericytes. In contrast, cefuroxime and vancomycin had minimal effects on cell viability within the concentration range used intraoperatively.

### Kinetics of toxicity of moxifloxacin, cefuroxime, and vancomycin determined by reducing ability

**Figure 2:**
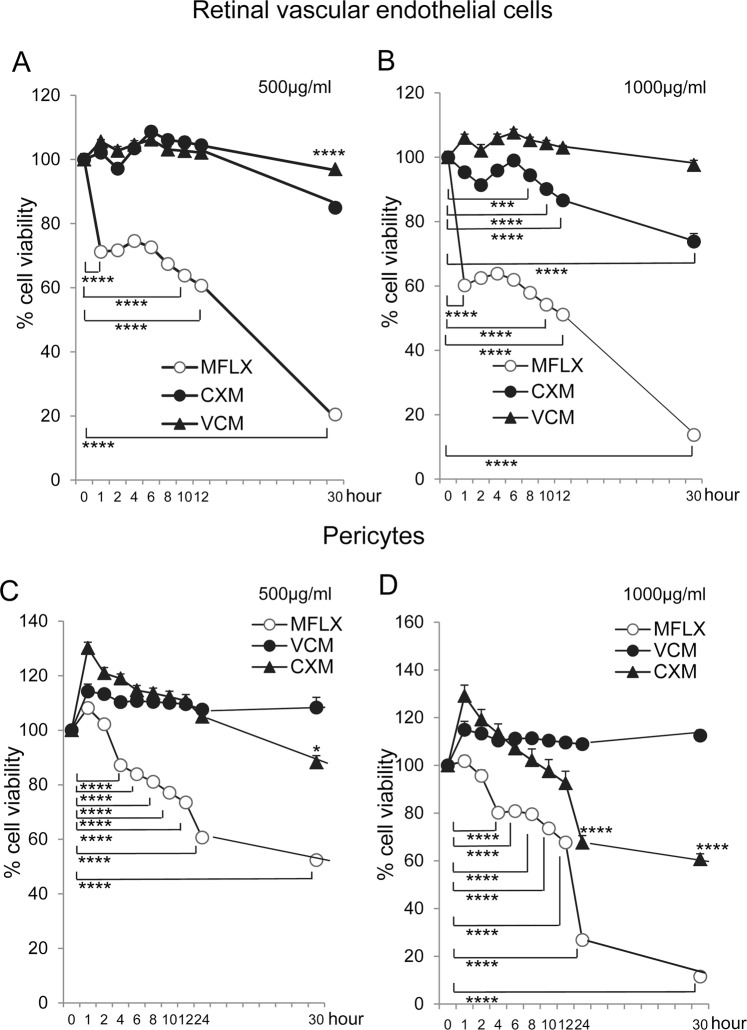
Kinetics of cell viability after exposure of human retinal vascular endothelial cells (RVECs) and pericytes to moxifloxacin, cefuroxime, or vancomycin. Cells were exposed to moxifloxacin, cefuroxime, or vancomycin at the indicated concentrations in 15% FCS-containing media for up to 30 h. The intrinsic reducing activity was used to assess the cell viability by chemiluminescence and the activity is expressed as the percentage of the controls. (**A**) Loss of reducing activity of human RVECs by antibiotics at 500 μg/mL. (**B**) Loss of reducing activity of human RVECs by antibiotics at 1000 μg/mL. (**C**) Loss of reducing activity of pericytes by antibiotics at 500 μg/mL. (**D**) Loss of reducing activity of pericytes by antibiotics at 1000 μg/mL. *P < 0.05; **P < 0.001 by ANOVA and post-hoc test. N = 5. Similar results were obtained after repeated experiments.

**Figure 3:**
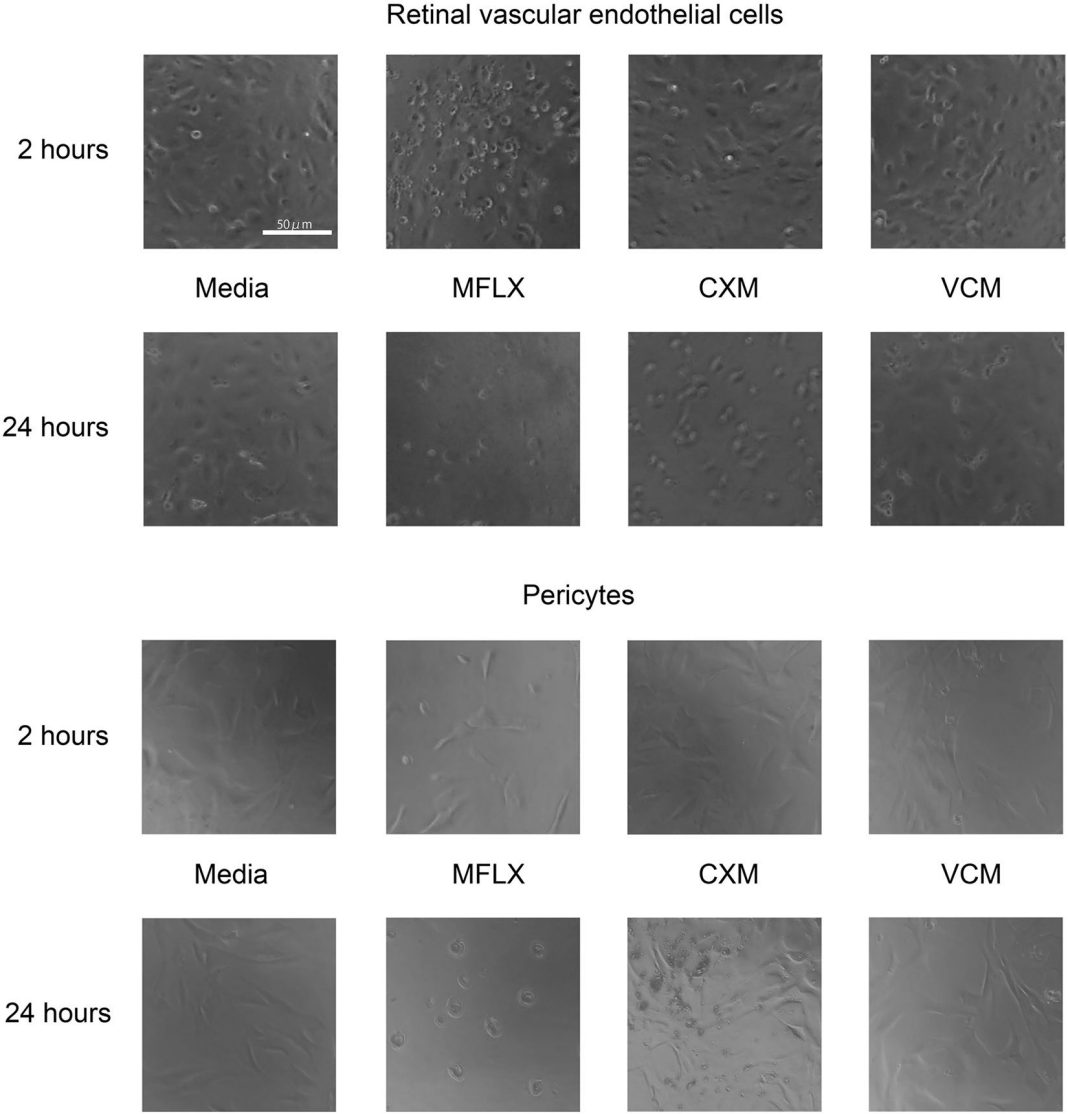
Cell morphological toxicities of moxifloxacin, cefuroxime, or vancomycin. Human retinal vascular endothelial cells (RVECs) and pericytes were exposed to Moxifloxacin (MFLX), cefuroxime (CXM), or vancomycin (VCM) at the indicated time points. Moxifloxacin-exposed RVECs began to round and detach as early as 2 h. They are severely damaged at 24 h. The cell rounding was observed at 30 h after cefuroxime exposure. Similar results were obtained after repeated experiments.

To determine the duration of exposure required to detect appreciable toxicity of the three antibiotics, we evaluated the intrinsic reducing activity of the vascular cells after their exposure to the antibiotics (Fig. 2). A depression of the reducing activity indicated a decrease of viability. For RVECs, moxifloxacin was found to induce a significant decrease in the reducing potential by 29% as early as 1 hour after the exposure (Fig. 2A,B). This effect was confirmed by a rounding of the cells and detachment from the plates (Fig. 3). These changes indicated significant alterations of the morphology of the cells even though the cells were not dead. The exposure to moxifloxacin induced a gradual decrease of cell viability, and the 50% survival time of reducing activity was 18 h. By 30 h of moxifloxacin exposure, most of the cells were dead (Fig. 2A,B).

In contrast, cells exposed to cefuroxime and vancomycin did not show any signs of toxicity for 1 h (Fig. 2A,B). At 30 h, cefuroxime (500 μg/ml) also induced a 20% decrease of reducing activity that was manifested by cell rounding (Fig. 3). Vancomycin did not have such toxic effects until 30 h.

Pericytes also showed impaired reducing potential by moxifloxacin (Fig. 2C,D). This effect was observed at 4 h after exposure, and the reducing potential decreased by 13% (moxifloxacin: 500 μg/ml). This effect was again confirmed by cell rounding and detachment. The exposure to moxifloxacin induced a gradual decrease of cell viability. The 50% survival time of reducing activity after exposure of 1000 μg/ml was 16 h (Fig. 2D). By 30 h of moxifloxacin exposure (1000 μg/ml), most of the cells were dead.

Pericytes exposed to cefuroxime or vancomycin did not show any signs of toxicity for 1 h (Fig. 2C,D). At 24 h, cefuroxime (1000 μg/ml) also induced a 32% decrease of its reducing activity.

### Induction of IL-8 in vascular endothelial cells by cefuroxime and vancomycin

Innate immune responses are activated through many different routes when cells are infected, injured, or under cellular stress^13^. Because the activation of the innate immune responses induces inflammatory cytokines, including interleukin-1β (IL-1β), interleukin-6 (IL-6), interleukin-8 (IL-8), and vascular endothelial growth factor (VEGF), we examined whether exposure to moxifloxacin, cefuroxime, or vancomycin will trigger the production of these cytokines.

**Figure 4:**
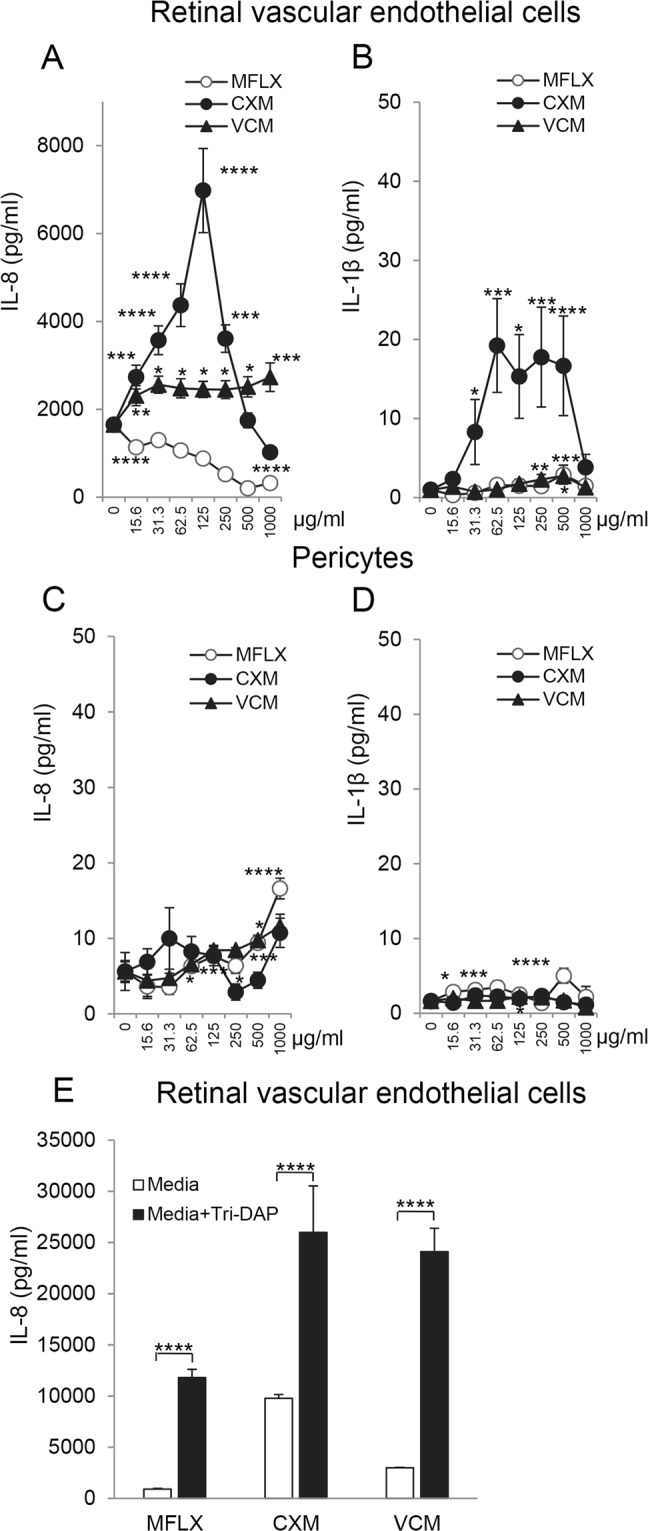
Induction of IL-8 and IL-1β secretion by human retinal vascular endothelial cells (RVECs) after exposure of vancomycin or cefuroxime. RVECs or pericytes were exposed to moxifloxacin, cefuroxime, or vancomycin for 24 h, and supernatants were measured for IL-8 and IL-1β by ELISA. (**A**) IL-8 secretion by RVECs after exposure of antibiotics for 24 h. (**B**) IL-1β secretion by RVECs after exposure of antibiotics. (**C**) IL-8 secretion by pericytes after exposure of antibiotics. (**D**) IL-1β secretion by pericytes after exposure of antibiotics. (**E**) Increased IL-8 secretion by RVECs by addition of Tri-Dap (1 µg/ml) exposure for 24 h. *P < 0.05; **P < 0.01; ***P < 0.005, ****P < 0.001 by ANOVA and post-hoc test; N = 5. Similar results were obtained after repeated experiments.

Our results showed that RVECs induced a significant level of IL-8 and IL-1β (Fig. 4A,B) exposure to the antibiotics, however no significant induction was observed for IL-6 or VEGF (data not shown). Constitutively, RVECs secrete IL-8 abundantly without stimulation. This IL-8 was significantly stimulated by exposure to 125 μg/mL of cefuroxime reaching almost 10000 pg/ml. Vancomycin exposure also significantly stimulated IL-8. Moxifloxacin exposure decreased the IL-8 production, presumably reflecting a reduction of cell viability. We also tested whether bacterial contaminants will exacerbate IL-8 production. When RVECs were treated with antibiotics in the presence of L-alanyl-γ-D-glutamyl-meso-diaminopimelic acid (Tri-DAP), degradative products of bacterial cell wall, IL-8 secretion was significantly elevated (Fig. 4E).

IL-1β secretion was also induced in RVECs after antibiotics treatment (Fig. 4B). All three antibiotics induced a significant increase in IL-1β. The induction by cefuroxime was most significant and was observed at ≥31.25 μg/mL. However, only small amount of IL-1β was secreted compared to the abundant levels of IL-8.

When pericytes were examined for IL-8 secretion (Fig. 4C), moxifloxacin and vancomycin induced IL-8, however the secreted IL-8 levels remained within 20 pg/ml and were almost negligible compared to those by RVECs. IL-1β was also induced by pericytes after exposure to moxifloxacin or vancomycin (Fig. 4D), however the induced levels were again limited.

### *In vivo* toxicity of intravitreal antibiotics

**Figure 5:**
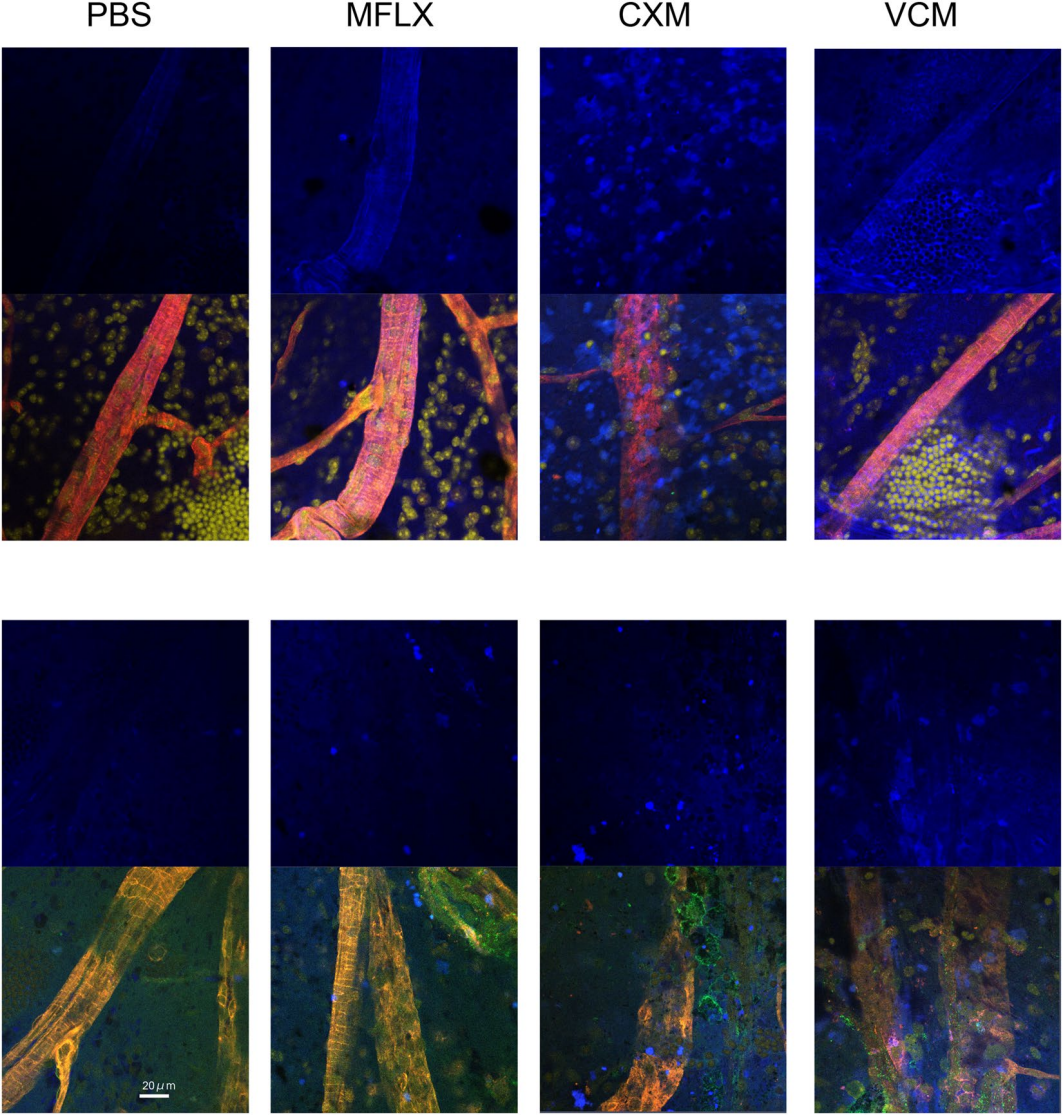
Retinal toxicity of moxifloxacin, cefuroxime, or vancomycin *in vivo*. Mice were injected intravitreally with moxifloxacin (MFLX), cefuroxime (CXM), or vancomycin (VCM), and assessed for retinal toxicity after 12 h using LIVE/DEAD Dead Cell Staining assay. Dead cell was stained blue (upper panel). Vascular endothelial cells were labeled by DyLight 594 conjugated-isolectin B4 (red). Pericytes were labeled by Alexa Fluor 488-conjugated NG2 antibody (Green, lower panel). Nucleus was stained by TO-PRO-3 iodide (yellow). Extensive toxicity was observed after the injection of cefuroxime or vancomycin with damaged cells extending to vascular endothelial cells and inner nuclear layer cells. In contrast, the toxicity of moxifloxacin was observed for only the retinal vascular endothelial cells.

To examine toxicity of the antibiotics on the retina *in situ*, mice were injected intravitreally with moxifloxacin, cefuroxime, or vancomycin and stained for cell membrane damage after 12 h. Notable retinal toxicity was observed after injection of cefuroxime or vancomycin (Fig. 5). Both antibiotics induced extensive cell membrane damage of the inner nuclear layer cells. Retinal vascular cells, including pericytes, were also damaged. In contrast, moxifloxacin-induced toxicity was confined to retinal vascular cells.

## Discussion

HORV and cefuroxime-associated hemorrhagic retinal infarction have been reported after intracameral antibiotics^14^. However, the mechanism(s) causing such complications has not been determined. HORV is characterized by sectorial hemorrhaging in non-perfused areas, macular ischemia, sectorial retinal vasculitis, and a rapid progression of neovascularization^15^. These characteristics strongly suggest that the pathological features are associated with retinal vascular damage caused by cefuroxime or vancomycin. Therefore, we examined whether the toxic and inflammatory effects of intracameral antibiotics are associated with a breakdown of the retinal vascular system. This was important because there is only limited information on the toxic effects of antibiotics on the retinal vascular endothelial cells.

A well-known adverse effect of antibiotics is their dose-dependent toxic effects which are caused by direct toxicity to individual cells. The direct cell damaging effect has been observed with exposure to moxifloxacin for both of RVECs and pericytes. Moxifloxacin also had direct toxic effects to other ocular cells. For example, corneal endothelial cells exposed to moxifloxacin caused cell damage with a reduction of intrinsic esterase activity^16^. An intracamerally injection of moxifloxacin can cause oxidative stress and caspase activation in corneal cells^17^. An intravitreal injection of 320 μg/0.1 ml of moxifloxacin, an intravitreal concentration of approximately 213 μg/ml, also caused a decrease of the ERGs, vacuolization of retina, and outer segment clumping^12^. A non-toxic dose was suggested to be 107 μg/ml (160 μg/0.1 ml) of moxifloxacin^12^. Our results showed that exposure of RVECs to >500 μg/ml of moxifloxacin caused significant damage and even death of RVECs. *In vivo*, the cellular damage will lead to a breakdown of the blood-retinal barrier. Moxifloxacin is also toxic to the trabecular meshwork cells and retinal pigment epithelial cells with an IC_50_ of 350 μg/mL^18,19^.

Vancomycin is known to be toxic to renal cells, and the toxicity has been attributed to oxidative stress^20^. Cefuroxime is also known to cause RPE damage at a concentration of 750 μg/ml for 24 h. Hemorrhagic retinal infarction, similar to HORV, was reported after an intracameral injection of cefuroxime following complicated cataract surgery^14^.

Our results showed that moxifloxacin could cause severe damage to RVECs and pericytes in culture while vancomycin and cefuroxime cause much less toxic damage. However, both cefuroxime and vancomycin can stimulate RVECs to secrete abundant amounts of IL-8, which can lead to an intense activation of innate or sterile inflammatory responses. IL-1β, which is induced by activated inflammasome upon cellular damage, was also stimulated by cefuroxime-treated RVECs.

Interestingly, intravitreal injection of the antibiotics led to extensive cellular damage *in vivo*, which was notable for cefuroxime and vancomycin. These antibiotics induced cell membrane damage of the ganglion cells and the inner nuclear layer. Because cell damage can release damage-associated molecular patterns (DAMP) including DNA, a DAMP-induced inflammasome activation will ensue. Thus, the cefuroxime and vancomycin-induced cell damage will cause stress responses and inflammatory cytokine secretion in these retinal layers. In contrast, moxifloxacin-induced cellular damage was limited to vascular cells.

We showed that exposure of RVECs to cefuroxime and vancomycin induces abundant IL-8 secretion reaching almost 10000 pg/ml. IL-8 is a very potent pro-inflammatory chemokine, and signals through CXCR1/CXCR2. IL-8 activates neutrophils, monocytes, and lymphocytes and also stimulates the recruitment of inflammatory cytokines including TNF-α. IL-8 is also vascular reactive, and it can increase the vascular endothelial permeability leading to vascular inflammation.

In the retina, the important CXCR1/CXCR2-bearing cells are the glial cells including the Müller cells, astrocytes, and microglial cells^21^. Müller cells are the principal microglial cells, and they span the entire retina from the internal limiting membrane to the subretinal space. The Müller cells contact the retinal cells at different levels and contribute to the inner blood:retinal barrier and the survival of neurons and photoreceptors. Astrocytes in the ganglion cells layer contact the blood vessels, and play crucial roles in the inner blood:retinal barrier together with the pericytes and the endothelial cells. Inflammatory stimuli, including IL-8, can impair the physiological functions of all these retinal glial cells in a prolonged manner. IL-8 can further stimulate the secretion of IL-8 and TNF-α by CXCR1/CXCR2 bearing cells^22^. Importantly, we found that vascular endothelial cells are a crucial source of IL-8 which may explain the delayed breakdown of retinal blood vessels and visual field loss which is observed in HORV.

HORV is very rare retinal disorder. Considering the worldwide prophylactic use of intracameral antibiotics during cataract surgery, the antibiotics are probably not the sole cause of HORV. Because of its rarity, the time of onset and repeatability suggested that the mechanism is a type 3 hypersensitivity reaction by the immune complex. However, no definitive proof of vancomycin-associated immunoglobulin complex has been detected.

One explanation is a contamination by gram positive bacteria-related products in rare and unexpected settings. Contamination can occur from the ocular surface flora or the surgical instruments. For example, numerous gram-positive bacteria including *corynebacterium* are present on the ocular surface. Moreover, contamination of surgical instruments can occur by the bacillus species. Of the bacterial products, Tri-DAPs are degenerative products of bacterial cell walls, and they are released when peptidoglycan synthesis is disrupted. Tri-DAP is typically contained in *Corynebacterium* and bacillus species and are resistant to heat sterilization. Thus, contaminated Tri-DAP can enhance the secretion of IL-8 from the vascular endothelial cells to further exacerbate the inflammatory responses. The genetic background of the patients may also be a prerequisite because some patients can develop HORV in both eyes.

The toxic effects of some types of drugs can be manifested as hypersensitivity or allergic reactions. For vancomycin, immune thrombocytopenia is a known rare complication. This is induced by the activation of vancomycin-dependent antiplatelet antibodies^23^. Generally, IL-1β also plays pivotal roles in activating B cells to secrete antibodies. Vancomycin can stimulate monocytes to induce toll-like receptors 1, 2, 4, 6, and 7^24^ which can recognize PAMP of bacteria. Because vancomycin inhibits the synthesis of bacterial cell walls, PAMP including Tri-DAP, can also be released in large quantities by disruptions of bacterial cell walls. HORV occurs a few days or a week after the intracameral application of vancomycin or cefuroxime and may fall into this category.

Our findings indicate that intracameral moxifloxacin needs to be used with greater cautious. Currently, the clinical dosage of moxifloxacin ranges from 100 to 1700 μg/mL in the anterior chamber, and the recommended intracameral dose is 500 μg/mL^25–29^. When directly injected into the vitreous cavity, the concentration is reduced to 7.5 to 127.5 μg/ml with a vitreous volume is 4 ml. Although higher concentrations may have some toxic effects, there appears to be no apparent side effects reported as moxifloxacin-related toxicity^25–29^.

However, toxicity of moxifloxacin appears transient and do not appreciably enhance the toxic inflammatory responses. To understand the drug-induced tissue damage, the clearance of the drug is important, and the clearance can be affected by various factors including the ionic properties, lipid solubility, molecular weight of the antibiotics, and presence of ocular inflammation^30^. High molecular weight cationic antibiotics, including vancomycin, are mainly cleared from the anterior chamber by diffusion or through Schlemm’s canal. The half-life of vancomycin in the vitreous cavity is very long and can reach 25.5 to 56 h. When 1000 μg/0.1 ml of vancomycin is injected intravitreally, the vitreal concentration is diluted to 250 μg/ml by the vitreous. This reduced concentration is very close to the concentration that can provoke inflammatory response and IL-8 secretion in the presence of Tri-DAP. Beta-lactams also have long half-life, and the half-life of ceftazidime is estimated to be 13.8 h in rabbit eyes^31^. In contrast, fluoroquinolones are cleared more readily from the vitreous cavity through active transport by the retinal capillaries and RPE cells. Moxifloxacin is cleared especially rapidly from the vitreous, and its estimated half-life is 1.72 h^32^. Considering the bactericidal effect, moxifloxacin is concentration-dependent, and the effects of the beta-lactams are time-dependent. Together, these properties and lower rates of endophthalmitis following their prophylactic use during cataract surgery may explain why the toxicity of moxifloxacin has not been reported.

Widely used perioperative antibiotic treatments during cataract surgery are usually topical and intracameral. The results of a recent meta-analysis showed that intracameral cefuroxime with or without topical levofloxacin lower the incidence of endophthalmitis^9^. This indicates that intracameral use of antibiotics should be continued. However, the choice of a specific antibiotic depends on the sensitivity profile of the organism. Surgeons may need to choose other antibiotics, such as moxifloxacin, based on commonly detected infectious species and drug resistance profile in the community. When choosing intracameral antibiotics for prophylaxis, it is important to understand the toxic profile of antibiotics on the retinal vasculature. Clinicians need to be aware that mistakes in the dilution or even direct diffusion into the vitreous cavity in aphakic eyes can have devastating outcomes.

There are several limitations in this study. Our analysis was performed on isolated retinal vascular cells and intravitreal injection of high concentration of antibiotics into mice eye, and may not duplicate the surgical setting of the retinal vasculature in the clinic. Thus, we need to be cautious that a safe concentration may be under or over estimated to avoid toxicity to the retinal vasculature.

In conclusion, moxifloxacin is directly toxic to retinal vascular cells at high doses. In contrast, vancomycin and cefuroxime can elicit strong inflammatory response as IL-8 secretion from retinal vascular endothelial cells dose dependently. Together with the strong toxicity of cefuroxime or vancomycin extending to the retinal inner nuclear layer and retinal vessels by injection, their administration may enhance innate or inflammatory responses presumably in sensitized subjects. Surgeons need to be cautious in choosing intracameral antibiotic prophylaxis as well as treating bacterial endophthalmitis.

## Methods

### Cells

Primary cultures were created from human retinal vascular endothelial cells isolated from human retinas (Cell Systems, Kirkland, WA). The RVECs were propagated to confluence on gelatin-coated 96-well plates in Dulbecco’s modified Eagle’s medium (DMEM; Gibco, Grand Island, NY) supplemented with 10% fetal bovine serum, L-glutamine, endothelial cell growth supplement (Sigma, St. Louis, MO), heparin, and non-essential amino acids (GIBCO).

### Measurements of membrane damage and cell viability

The RVECs (1 × 10^4^ cells/well) were grown in 96-well plates and exposed to serially-diluted moxifloxacin (Vigamox^®^, Alcon, Fort Worth, TX), cefuroxime (GlaxoSmithKline, Brentford, UK), or vancomycin (Meiji, Tokyo, JPN) in serum-depleted medium for 24 hours. The cell membrane damage caused by these antibiotics allowed ethidium homodimer-1 (EthD-1; Molecular Probes, Eugene, OR) to enter the cells and bind to the DNA. To determine the degree of damage, the amount of bound EthD-1 was measured with a fluorescent microplate reader (Tecan, Männedorf, Switzerland) with excitation by 495 nm light and emission at 635 nm. Saponin (0.1%, Sigma, St. Louis, MO)-treated RVECs were used as positive control of dead cells.

We measured the cell viability by the esterase activity because viable cells have intrinsic esterase activity. To measure the esterase activity, antibiotics-exposed RVECs were exposed to non-fluorescent calcein AM (Molecular Probes, Eugene, OR), which is converted to fluorescent calcein by the esterase activity of living cells. The intensity of the fluorescence was measured with a microplate reader with excitation by 495 nm and emission at 530 nm.

The cell viability and membrane damage were calculated by the following formula, % alteration = (antibiotics-treated cell emission – non-treated cell emission)/(Positive control cell emission – non-treated cell emission) × 100.

### Kinetics of cell viability

RVECs (1 × 10^4^ cells/well) were grown on 96-well plates and exposed to cefuroxime, vancomycin, or moxifloxacin in 10% FBS supplemented media. To measure the cell viability, the intrinsic reducing ability of the metabolically active cells was measured by the MT cell viability assay (RealTime-Glo, Promega, Madison, WI) after the antibiotics exposure.

### Measurements of osmolarity of media with antibiotics

The osmolarity of the media was measured with a freezing point depression osmometer (Osmomat 3000, Gonotec, Germany). The osmolarity of the media with moxifloxacin, cefuroxime, or vancomycin (2000 μg/ml) was 306 mOsmol/Kg, 332 mOsmol/Kg, and 326 mOsmol/Kg, respectively. The osmolarity of the medium without antibiotics was 320 mOsmol/Kg.

### Enzyme-linked immunosorbent assay (ELISA)

The supernatants of the RVECs exposed to the antibiotic with or without Tri-DAP (InvivoGen, San Diego,CA,1 μg/ml) were collected after 24 h, and the level of interleukin (IL-8) and interleukin-1β (IL- β) was measured with commercial ELISA kits (BioLegend, San Diego, CA). The supernatant was diluted 5-fold with the diluent of the kit, and the mixture was incubated on the antibody-coated plates overnight at 4 °C. The plates were processed for ELISA using the intensity of chemiluminescence to determine the level of IL- β.

### *In vivo* toxicity assay of intravitreal antibiotics

Seven-week-old normal C57BL/6 mice were intravitreally injected with 2 μl of moxifloxacin, cefuroxime, or vancomycin (2000 μg/ml). After 12 h, anesthetized mice were perfused with PBS followed by a systemic perfusion of 10 ml of the working solution of LIVE/DEAD Fixable Dead Cell Stain kit (Invitrogen, Waltham, MA). The eyes were enucleated, and the retinas were flat mounted. The flat mounts were washed with PBS, fixed with 4% paraformaldehyde, and stained with DyLight 594 conjugated-isolectin B4 (Vector Laboratories, Peterborough, UK) for endothelial cells, Alexa Fluor 488-conjugated anti-NG2 antibody for pericytes (Sigma), and TO-PRO-3 iodide (Molecular Probes, Waltham, MA) for nuclear staining. The retinas were examined and photographed with a confocal microscope (LSM730, Zeiss, Germany).

All mice were treated in accordance with the ARVO Statement for the Use of Animals in Ophthalmic and Vision Research and protocols approved by the Institutional board of Animal Care and Use Committee of Tottori University.

### Statistical analyses

Data are presented as the means ± standard error of the means (SEMs). The significance of the differences was determined by *t* tests or ANOVA and post hoc tests. A *P*-value < 0.05 was taken to be statistically significant.
