# Publisher Correction: Toxicities of and inflammatory responses to moxifloxacin, cefuroxime, and vancomycin on retinal vascular cells

**DOI:** 10.1038/s41598-020-75486-8

**Published:** 2020-10-23

**Authors:** Hitomi Miyake, Dai Miyazaki, Yumiko Shimizu, Shin-ichi Sasaki, Takashi Baba, Yoshitsugu Inoue, Kazuki Matsuura

**Affiliations:** Division of Ophthalmology and Visual Science, Faculty of Medicine, Tottori University, Department of Ophthalmology, Nojima Hospital, Tottori, Japan

Correction to: *Scientific Reports* https://doi.org/10.1038/s41598-019-46236-2, published online 05 July 2019.

In the original version of this Article, Dr Kazuki Matsuura was incorrectly listed as the corresponding author. The correct corresponding author for this Article is Dr Dai Miyazaki.

Correspondence and request for materials should be addressed to miyazaki-ttr@umin.ac.jp. This error has now been corrected in the HTML and PDF version of the Article.

